# The effect of ciprofol on the incidence of postoperative delirium in adult surgical patients: a meta-analysis and meta-regression

**DOI:** 10.3389/fneur.2026.1740470

**Published:** 2026-02-04

**Authors:** Pengxue Guo, Xinmin Li, Chunlin Ren, Zhenfei Duan, Yuting Kong, Mengyao Bi, Feizhou Liu, Ye Wang, Lidian Chen, Yasu Zhang

**Affiliations:** 1 Rehabilitation Medicine College, Henan University of Chinese Medicine, Zhengzhou, China; 2 School of Traditional Chinese Medicine, Henan University of Chinese Medicine, Zhengzhou, China

**Keywords:** anesthesia, ciprofol, meta-analysis, meta-regression, postoperative delirium

## Abstract

**Objective:**

To systematically evaluate the impact of ciprofol on the incidence of postoperative delirium (POD) in adult surgical patients and to explore potential sources of heterogeneity.

**Methods:**

A systematic search was conducted in databases including PubMed, Web of Science, OVID, EMBASE, and the Cochrane Library to identify clinical studies published before October 2025 comparing ciprofol vs. propofol for general anesthesia. The Newcastle-Ottawa Scale was employed to assess study quality. Meta-analysis and meta-regression were performed using R software to calculate the POD incidence and its 95% confidence interval (CI). Subgroup analysis and sensitivity analysis were conducted to explore sources of heterogeneity.

**Results:**

Seven studies involving 4,171 patients were included. The overall POD incidence in the ciprofol group was 11.30% (95% CI: 0.77%−21.83%), which was significantly lower than that in the propofol group (19.51%; 95% CI: 2.51%−36.50%). Subgroup analysis revealed that the advantage of ciprofol in reducing POD incidence was more pronounced in patients undergoing trunk surgery (19.29% vs. 0.56%) and in those receiving total intravenous anesthesia (2.93% vs. 14.33%). Meta-regression did not identify significant correlations between POD incidence and age, sex distribution, or intraoperative hypotension. Significant heterogeneity was observed across studies (*I*^2^ > 85%), but sensitivity analysis confirmed the robustness of the results.

**Conclusion:**

Compared with propofol, ciprofol significantly reduces the risk of POD in surgical patients, with particularly pronounced benefits in those undergoing trunk surgery and receiving total intravenous anesthesia. These findings provide new evidence for perioperative neurocognitive protection.

**Systematic Review Registration:**

INPLASY International Platform, registration number: INPLASY202610019, DOI: 10.37766/inplasy2026.1.0019.

## 1 Introduction

Postoperative delirium (POD) is a common and serious complication in surgical patients, characterized by acute and fluctuating disturbances in attention, awareness, cognition, and perception (1). POD not only significantly prolongs hospital stay and increases healthcare costs but is also closely associated with higher mortality, long-term cognitive decline, and increased dependence on rehabilitation facilities after discharge. Consequently, exploring effective perioperative intervention strategies to reduce the incidence of POD has become a crucial research focus in modern anesthesiology and perioperative medicine (2).

The choice of anesthetic agent is considered one of the key modifiable factors influencing the occurrence of POD (3). Currently, intravenous anesthetics, particularly propofol, are central to both the induction and maintenance of general anesthesia. However, the clinical use of propofol has certain limitations, including injection pain, respiratory and cardiovascular depression, and the risk of hyperlipidemia, all of which may potentially adversely affect postoperative recovery, especially neurocognitive function (4). Ciprofol, a novel intravenous anesthetic, is the dextro-isomer of propofol and exerts its sedative-hypnotic effects by acting on the γ-aminobutyric acid type A (GABAA) receptor. Compared to propofol, ciprofol offers potential advantages such as rapid onset, fast metabolism, fewer adverse effects on the respiratory and cardiovascular systems, and a significantly lower incidence of injection pain (5). These improved pharmacological properties suggest that ciprofol may represent a more favorable anesthetic option for enhanced recovery after surgery, particularly in terms of neurofunctional protection.

In recent years, several randomized controlled trials have begun to investigate differences in POD incidence between ciprofol and propofol. However, existing studies are generally limited by small sample sizes, and their conclusions are inconsistent. Some studies report a significantly lower incidence of POD in patients receiving ciprofol compared to those receiving propofol, while others observed no statistically significant difference (6). This inconsistency may stem from insufficient statistical power in individual studies, heterogeneity in patient populations (e.g., age, surgical type, baseline cognitive status), or subtle variations in anesthetic management protocols.

Given this background, there is a pressing need for a comprehensive quantitative analysis to synthesize the available evidence, thereby providing a more precise and reliable assessment of the effect of ciprofol on POD. Meta-analysis serves as a powerful statistical tool that can enhance sample size and increase statistical power by pooling results from multiple independent studies, leading to more generalizable conclusions (7). Furthermore, meta-regression can be employed to explore potential sources of heterogeneity among study findings.

Therefore, this study aims to conduct a systematic review and critical appraisal of the existing literature to perform a meta-analysis and meta-regression, with the following core objectives: to systematically evaluate the impact of ciprofol vs. propofol on the incidence of postoperative delirium in adult surgical patients, and to explore potential sources of heterogeneity—including patient age, surgical type, and anesthesia technique—via subgroup analysis and meta-regression.

The findings of this study will provide high-level evidence-based medical evidence to assist clinicians in selecting optimal anesthetic regimens during the perioperative period to improve patient outcomes, particularly in the prevention of POD.

## 2 Method

### 2.1 Inclusion and exclusion criteria

The inclusion criteria were as follows: (1) surgical patients undergoing general anesthesia; (2) studies in which ciprofol was utilized as the primary anesthetic agent; (3) studies that assessed the incidence of POD in patients anesthetized with ciprofol; (4) randomized controlled trials (RCTs).

The exclusion criteria were as follows: (1) literature reviews, case reports, commentaries, letters, and conference abstracts; (2) duplicate publications or studies with overlapping patient populations; (3) studies not published in English; (4) studies that did not report or from which data on delirium events in surgical patients receiving ciprofol anesthesia could not be extracted.

### 2.2 Search strategy

Given the limited volume of literature in this specific field, the search strategy was designed using “Ciprofol” as the primary search term, incorporating MeSH terms and other relevant keywords. Key search terms included “Ciprofol,” “HSK3486,” and “2-((1R)-1-cyclopropyl)ethyl-6 -isopropyl-phenol” which were combined using Boolean operators. A systematic search was conducted across authoritative databases, including PubMed, Web of Science, OVID, EMBASE, and the Cochrane Library. Retrieved records were imported into EndNote 2025 for management. Two researchers independently performed an initial screening of the identified articles based on the relevance of their references. Any discrepancies encountered during the article selection process were resolved through consultation with a third investigator. The search was updated until October 2025.

### 2.3 Data extraction

Two investigators independently conducted a full-text review and extracted data from studies meeting the inclusion criteria. A standardized data extraction form was developed to collect information, including the first author, publication year, diagnostic criteria for POD, patient age, sex, follow-up duration, country or region, number of enrolled patients, incidence of POD, as well as surgical type and anesthesia technique. Disagreements during data extraction were resolved through discussion and consensus. If consensus could not be reached, a third researcher made the final determination.

### 2.4 Quality assessment

The methodological quality of the included randomized controlled trials was assessed using the Cochrane Risk of Bias tool version 2 (RoB 2). This tool evaluates five domains: randomization process, deviations from intended interventions, missing outcome data, measurement of the outcome, and selection of the reported result. Each study was subsequently classified as having “low risk,” “some concerns,” or “high risk” of bias. Two investigators independently performed the assessments, and any discrepancies were resolved through discussion or consultation with a third investigator.

### 2.5 Data analysis

Meta-analysis was performed using the “meta” and “metafor” packages in R software (version 4.2.2) (8). Raw incidence data were first subjected to logarithmic, logit, arcsine, and Freeman-Tukey double arcsine transformations. The normality of the distribution was assessed using the Shapiro-Wilk test, and the most appropriate transformation method was selected based on the results. The incidence of POD in surgical patients receiving ciprofol anesthesia, along with its 95% confidence interval (CI), was calculated. Heterogeneity among the included studies was evaluated using Cochrane's *Q*-test and the *I*^2^ statistic. Significant heterogeneity was considered present if the *p*-value from Cochrane's *Q*-test was ≤ 0.05 or if *I*^2^≥ 50%. In such cases, subgroup analysis was employed to observe whether heterogeneity decreased within specific subgroups. Sensitivity analysis was conducted by iteratively omitting individual studies to assess the robustness of the pooled results. Studies identified as exerting an unduly influential effect on the analysis were considered for exclusion to determine if their removal resolved the heterogeneity.

If substantial heterogeneity persisted after these steps, a random-effects model was used to calculate the pooled incidence and its 95% CI, and sources of heterogeneity were further investigated. In the absence of significant heterogeneity, a fixed-effects model was applied to pool the overall incidence. Publication bias was assessed using funnel plots and Egger's test. If obvious outliers were identified, their potential sources of bias were carefully analyzed before considering exclusion. Meta-regression analysis was performed to examine the relationship between reported continuous variables and the incidence of POD.

The conduct of this meta-analysis adhered to the guidelines outlined in the Preferred Reporting Items for Systematic Reviews and Meta-Analyses (PRISMA) statement. The protocol for this systematic review and meta-analysis was registered on the INPLASY international platform (registration number: INPLASY202610019; doi: 10.37766/inplasy2026.1.0019).

## 3 Results

### 3.1 Characteristics and Quality of the Included Studies

**Figure 1:**
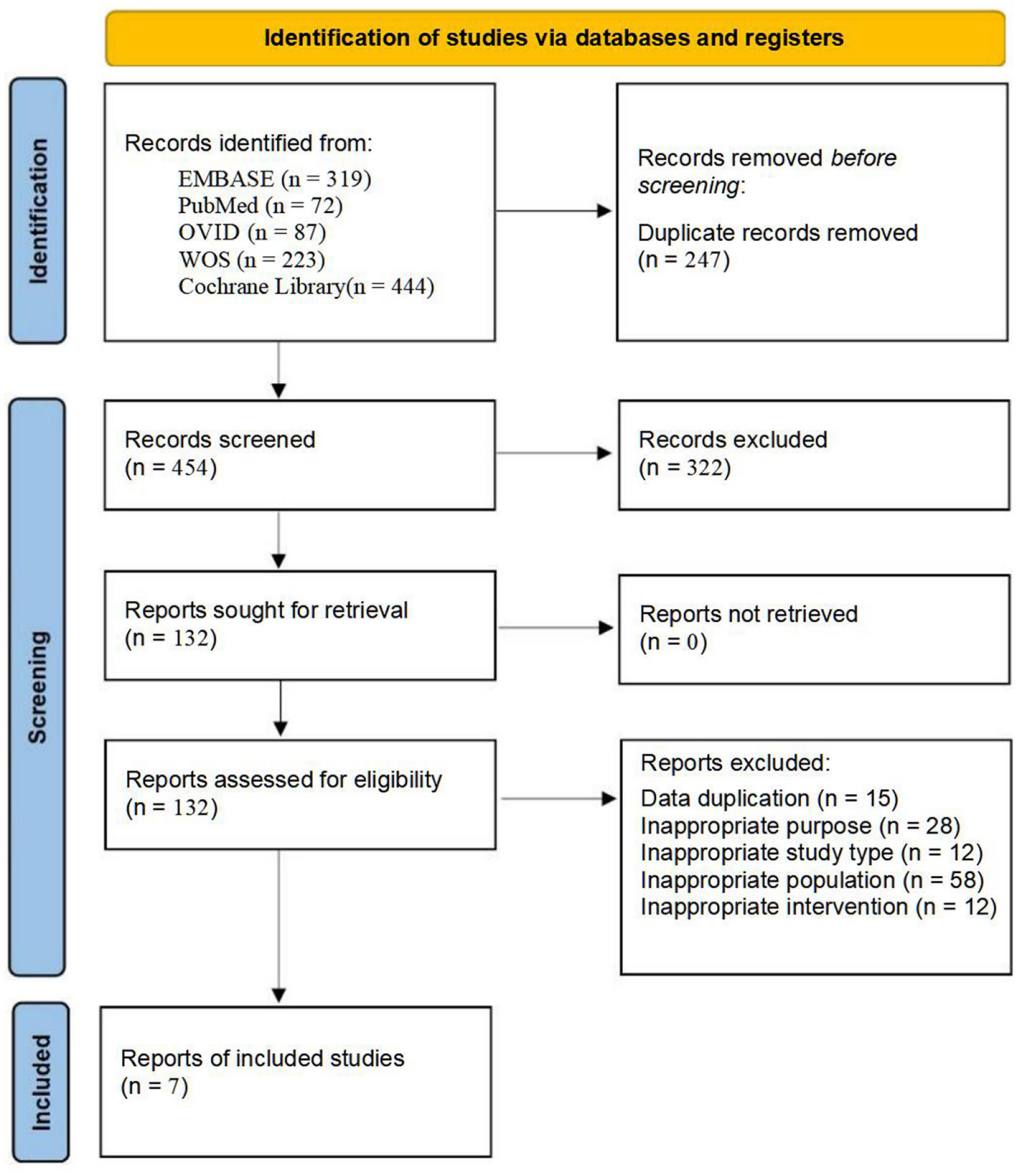
Flow chart of study selection process.

A total of 1,145 articles were initially identified through database searches. After preliminary screening and full-text assessment, seven studies were ultimately included for analysis (Table 1, Figure 1) (9–15). All included studies were published in English after 2020. These studies collectively involved 4,171 patients. Quality assessment conducted according to the RoB 2 standard indicates that all the studies met the requirements.

**Table 1:** Characteristics of studies included in the meta-analysis.

**Study **	**Surgery type **	**Intervention (Obs) **	***n* (Obs) **	**Intervention (Ctrl) **	***n* (Ctrl) **	**Mean age (Obs)**	**Mean age (Ctrl) **	**Age (range) **	**Male % (Obs) **	**Male % (Ctrl) **	**Preop cognitiveassess**	**Anesthesia **	**Delirium, *n* (Obs) **	**Delirium, *n* (Ctrl) **	**IO hypotension (Obs) **	**IO hypotension (Ctrl) **
Liu 2024	Thoracoscopic surgery for lung cancer	Ciprofol	42	Propofol	42	68.5	68	65–80	0.5000	0.4290	MMSE	TIVA	3	7	78.60%	85.70%
Zhang 2025	Fundus surgery	Ciprofol	30	NA	34/29	50.4	48.8	18–65	0.6000	0.4710	NA	TIVA	0	0	53.30%	38.20%
Shi 2025	Ureteroscopic surgery	Ciprofol	55	Propofol	54	56.3	55.1	18–75	0.5640	0.6110	NA	BIVA	0	0	32.70%	57.40%
Lu 2025	Cardiac surgery with cardiopulmonary bypass	Ciprofol	64	Propofol	65	54.5	55	≥18	0.4060	0.4770	MMSE	BIVA	19	34	18.75%	44.62%
Chen 2025	Hip surgery	Ciprofol	55	Propofol	55	61	62	NA	0.4910	0.5090	MMSE	NA	3	11	23%	42%
Di 2025	cardiac surgery	Ciprofol	50	NA	NA	69	NA	NA	0.7200	NA	MoCA-B	NA	19	N/A	NA	NA
Liang 2024	laparoscopic major abdominal surgery	Ciprofol	72	Propofol	72	68	68	NA	0.4720	0.5560	MMSE	TIVA	4	8	33.30%	54.20%

Obs, observation group; Ctrl, control group; MMSE, Mini-Mental State Examination; MoCA-B, Montreal Cognitive Assessment-Basic; TIVA, Montreal Cognitive Assessment-Basic; BIVA, Balanced Intravenous-Inhalational Anesthesia.

### 3.2 Incidence of delirium in surgical patients anesthetized with ciprofol

**Figure 2:**
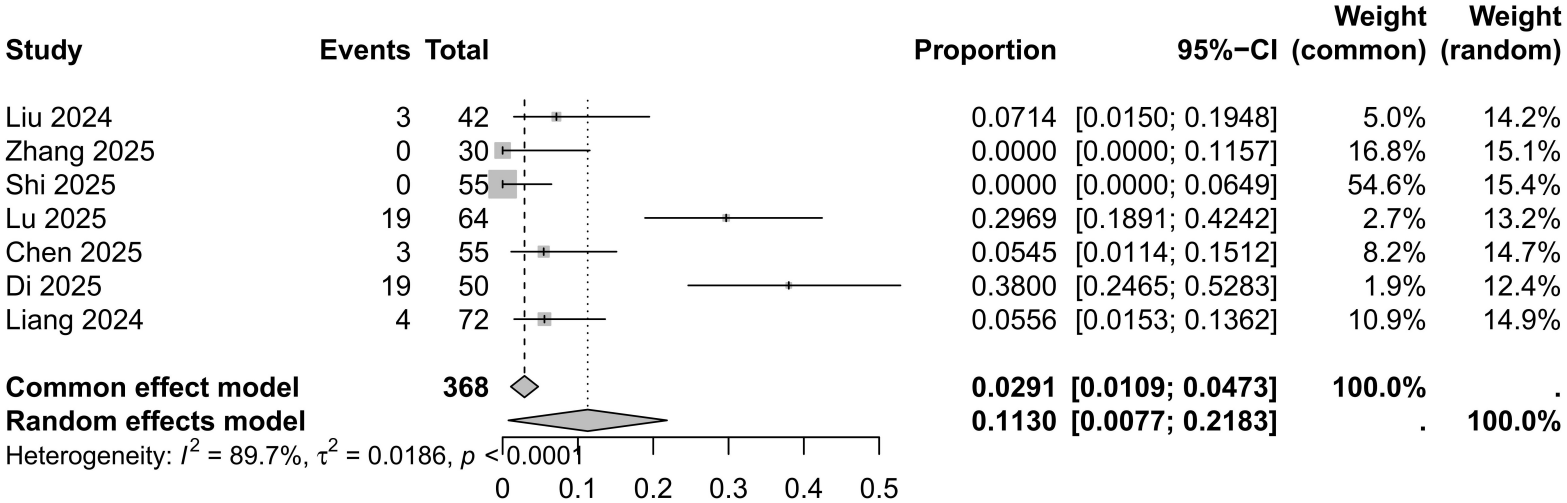
Forest plot for the incidence of postoperative delirium in patients anesthetized with ciprofol.

The raw data for the incidence of delirium in surgical patients receiving ciprofol anesthesia were normally distributed (*p* = 0.0274); therefore, the raw data were used directly in the meta-analysis. As shown in Figure 2, the pooled overall incidence was 0.1130 (0.0077; 0.2183) (*I*^2^ = 89.7%, *p* < 0.01), indicating significant heterogeneity among the included studies according to both Cochrane's *Q*-test and the *I*^2^ statistic. Funnel plot inspection and Egger's test (*t* = 3.34, *p* = 0.01) suggested potential publication bias; however, the robustness of this finding is limited due to the small number of studies available for the funnel plot. Sensitivity analysis performed by sequential omission of individual studies did not identify any single study exerting an undue influence on the overall results. Consequently, a random-effects model was adopted to present the pooled result.

### 3.3 Incidence of delirium in surgical patients anesthetized with propofol

**Figure 3:**
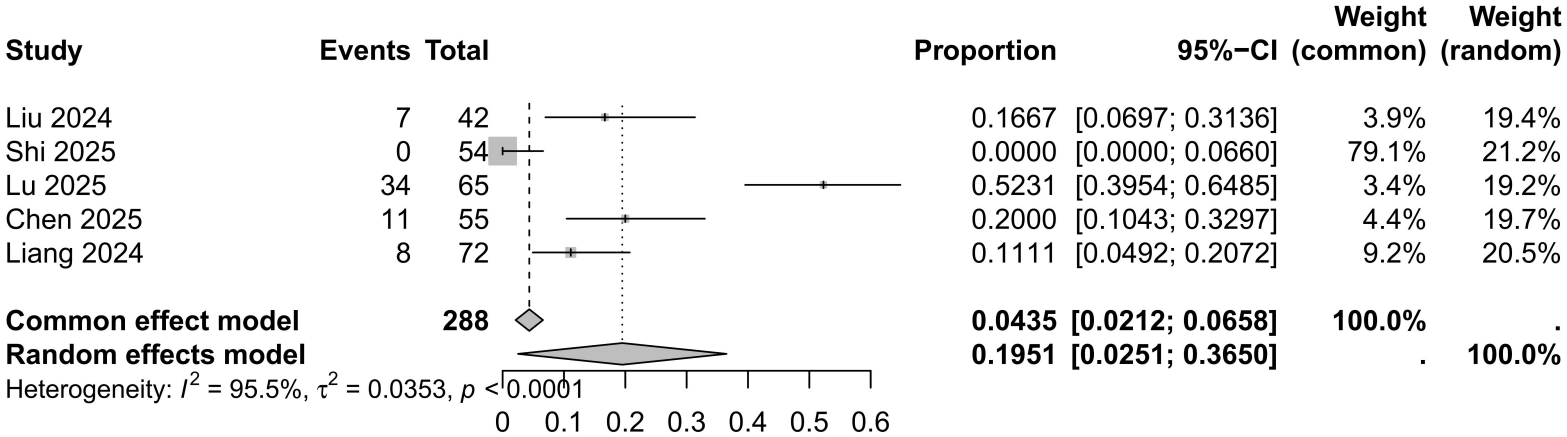
Forest plot for the incidence of postoperative delirium in patients anesthetized with propofol.

As five studies utilized propofol as the control intervention, a meta-analysis of the incidence of delirium in surgical patients receiving propofol anesthesia from these studies was also conducted. The pooled incidence in the propofol group was 0.1951 (0.0251; 0.3650) (*I*^2^ = 95.5%, *p* < 0.01; Figure 3). Significant heterogeneity was also observed among these studies based on Cochrane's *Q*-test and the *I*^2^ statistic. Due to the limited number of studies involving propofol, funnel plots and Egger's test were not performed. Sensitivity analysis via sequential omission identified no single study that disproportionately influenced the combined result. Thus, a random-effects model was employed to describe the pooled result.

### 3.4 Subgroup analysis

Subgroup analyses were conducted based on surgery type (trunk, encompassing major thoracic and abdominal procedures vs. peripheral), patient age (adults vs. elderly), and anesthesia technique (total intravenous anesthesia [TIVA] vs. balanced intravenous-inhalational anesthesia). The results were as follows: the pooled incidence of delirium in patients undergoing trunk surgery anesthetized with ciprofol was 0.1929 (0.0354; 0.3504) (*I*^2^ = 90.4%, *p* < 0.01), whereas it was 0.0056 (0.0000; 0.0260) (*I*^2^ = 21.5%, *p* = 0.28) in those undergoing peripheral surgery. The pooled incidence in elderly patients anesthetized with ciprofol was 0.1611 (0.0000; 0.3603) (*I*^2^ = 89.8%, *p* < 0.01), compared to 0.0801 (0.0000; 0.2096) (*I*^2^= 89.3%, *p* < 0.01) in adult patients. For patients receiving TIVA, the pooled incidence was 0.0293 (0.0000; 0.0612) (*I*^2^ = 43.9%, *p* = 0.17), while it was 0.1433 (0.0000; 0.4340) (*I*^2^ = 96.2%, *p* < 0.01) for those receiving balanced intravenous-inhalational anesthesia.

### 3.5 Sensitivity analysis

The sensitivity analysis demonstrated that sequentially omitting any single study from the various meta-analyses did not significantly alter the overall results. This indicates that the high degree of heterogeneity observed in the results was not driven by any individual study, suggesting other origins for the heterogeneity. Further analysis pertaining to this finding will be elaborated upon in the section 4.

### 3.6 Meta-regression

Meta-regressions were performed to assess the relationship between the incidence of postoperative delirium following ciprofol anesthesia and the covariates of mean age, incidence of intraoperative hypotension, and sex ratio (proportion of males).

The meta-regression of mean age on delirium incidence revealed that, despite high heterogeneity among studies (*I*^2^ = 85.83%), age did not explain this heterogeneity (*R*^2^ = 0.00%). No significant association was found between mean age and delirium incidence [β = 0.0626 (-0.1058, 0.2310), *p* = 0.4664], suggesting that current evidence does not support age as a predictor of postoperative delirium following ciprofol anesthesia.

The meta-regression of sex ratio on delirium incidence indicated that, despite high heterogeneity (*I*^2^ = 85.02%), sex ratio did not account for this heterogeneity (*R*^2^ = 0.00%). No significant association was observed between sex ratio and delirium incidence [β = 1.6839 (-9.4755, 12.8434), *p* = 0.7674], indicating that current evidence does not support sex ratio as a predictor of postoperative delirium following ciprofol anesthesia.

Six included studies reported the incidence of intraoperative hypotension. The meta-regression of intraoperative hypotension on delirium incidence showed that, despite high heterogeneity (*I*^2^ = 78.18%), intraoperative hypotension did not explain this heterogeneity (*R*^2^ = 0.00%). No significant association was found between the incidence of intraoperative hypotension and the incidence of postoperative delirium following ciprofol anesthesia [β = −1.4718 (-6.4251, 3.4815), *p* = 0.5603], suggesting that current evidence does not support intraoperative hypotension as a predictor in this context.

## 4 Discussion

Through a systematic review and meta-analysis, this study provides the first comprehensive evaluation of the difference in the incidence of POD associated with ciprofol vs. propofol in adult surgical patients. The findings not only reveal a numerical difference in POD incidence between the two anesthetic agents but also deepen our multidimensional understanding of perioperative neurocognitive protection.

### 4.1 Principal findings and clinical implications

This study found that the overall pooled POD incidence in patients anesthetized with ciprofol was 0.1130, significantly lower than the 0.1951 observed in the propofol group. This finding carries considerable clinical significance. From a practical standpoint, a reduction in POD implies that a substantial number of patients could avoid the associated prolonged hospital stays, increased healthcare costs, and long-term cognitive decline. Given the established association between POD and increased 30-day mortality, the clinical use of ciprofol may yield substantial long-term benefits (16). From a health economics perspective, although the unit cost of ciprofol may be higher than that of propofol, its advantage in reducing POD incidence could lead to a more favorable cost-effectiveness profile by mitigating complication management costs and shortening hospitalization.

### 4.2 Exploration of pharmacological mechanisms

The potential superiority of ciprofol in POD prevention may stem from its distinct pharmacological properties. Firstly, ciprofol exhibits higher selectivity in activating the GABA-A receptor, potentially inducing different neurophysiological effects via modulation of specific receptor subtypes (17, 18). Secondly, its significantly reduced incidence of injection pain implies attenuated intraoperative stress response, which is closely linked to neuroinflammation (19). Furthermore, ciprofol's more stable hemodynamic profile helps maintain cerebral perfusion pressure, thereby avoiding neuronal injury secondary to cerebral hypoperfusion (20). Notably, ciprofol's metabolic characteristics may reduce drug accumulation in brain tissue, which could be a crucial factor facilitating rapid postoperative cognitive recovery (21).

### 4.3 Interpretation of subgroup analyses

The subgroup analyses offer valuable insights for precision anesthesia. Regarding surgery type, the significantly higher POD incidence in patients undergoing trunk surgery compared to peripheral procedures is likely related to the more pronounced systemic inflammatory response syndrome triggered by major surgery. Inflammatory cytokines such as IL-6 and TNF-α can cross the blood-brain barrier, activate microglia, disrupt neurotransmitter balance, and ultimately precipitate delirium (22). In this context, ciprofol may indirectly suppress the neuroinflammatory cascade by attenuating surgical stress response and maintaining physiological homeostasis (23).

Regarding anesthesia technique, the lower POD incidence in patients receiving TIVA compared to those under balanced intravenous-inhalational anesthesia aligns with growing evidence on the potential neurotoxicity of volatile anesthetics (24). Inhaled agents have been demonstrated to promote Aβ oligomerization, enhance Tau protein phosphorylation, and disrupt synaptic plasticity (25). The use of ciprofol within a TIVA protocol offers a dual advantage: avoiding the potential neurotoxicity of inhaled anesthetics while potentially leveraging any intrinsic neuroprotective properties of ciprofol itself.

The meta-regression did not identify significant associations between POD incidence and mean age, sex ratio, or intraoperative hypotension. However, these findings should be interpreted with caution due to the potential for ecological fallacy. Meta-regression operates on study-level aggregated data and cannot infer individual-level risk associations; it may also mask within-group variation. Moreover, the development of POD is multifactorial. Beyond the variables tested, factors such as preoperative cognitive function, duration of surgery, perioperative glycemic fluctuations, quality of pain management, and sleep disturbances may also play significant roles. Recent studies have also suggested that intraoperative end-tidal CO_2_ management, perioperative intravenous lidocaine, or glucocorticoid administration may influence neurocognitive outcomes. Future research should integrate these potential moderators into the analytical framework (26–28).

### 4.4 Reconsidering sources of heterogeneity

The substantial heterogeneity observed in this study (*I*^2^ > 85%) suggests that the effect of ciprofol on POD may be modulated by a range of clinical and methodological factors. In addition to the sources previously discussed, the following aspects may further explain this variability: first, differences in anesthesia depth management across study centers—such as the use and target ranges of monitors like the Bispectral Index—could significantly influence neurocognitive outcomes. Second, inconsistencies in postoperative analgesic regimens, particularly opioid consumption and the application of regional blockade techniques, may affect delirium occurrence through both pain control and drug-specific pathways. Furthermore, the varying implementation of institutional delirium prevention strategies, such as early mobilization, sleep protection, and cognitive stimulation, constitutes an important confounding factor. This diversity in clinical practice reflects real-world complexity and underscores the need for future studies to standardize perioperative management protocols more rigorously.

### 4.5 Methodological considerations and strength of evidence

This study was conducted in strict accordance with PRISMA guidelines, employing a comprehensive search strategy and standardized analytical methods. We performed multiple transformations of the incidence data to ensure robustness, and sensitivity analyses confirmed the stability of the pooled results.

However, significant limitations must be acknowledged. The included studies, while all RCTs, exhibited substantial and largely unexplained heterogeneity (*I*^2^ > 85%), and there was statistical evidence suggestive of publication bias. Given these constraints, the present evidence base should be considered preliminary and exploratory. The primary aim of this analysis was not to provide definitive conclusions for clinical practice, but rather to systematically synthesize the existing and inconsistent evidence, quantify the overall trend, and rigorously investigate potential sources of heterogeneity (e.g., surgery type, anesthetic technique). Therefore, a formal GRADE assessment of the certainty of evidence was not performed for the main finding, as applying a simplified classification (e.g., “low” or “very low”) to a quantitative result derived from such a heterogeneous and immature evidence pool could be potentially misleading. Instead, we have transparently detailed all methodological limitations through risk-of-bias assessment, heterogeneity quantification, subgroup analysis, meta-regression, and publication bias tests.

We emphasize that the current evidence on the effect of ciprofol on POD is nascent and of low certainty. The results presented here must be interpreted with utmost caution. They serve to generate hypotheses and highlight critical knowledge gaps, foremost being the need for future large-scale, rigorously designed RCTs with POD as a primary endpoint. A formal GRADE evaluation will be an essential step when such more definitive evidence becomes available.

### 4.6 Directions for future research

Based on the findings and limitations of this study, future research should focus on the following directions: firstly, there is a need for large-scale RCTs designed with POD as a primary endpoint, employing uniform delirium assessment tools and timepoints to ensure result comparability. Secondly, studies targeting specific populations (e.g., advanced age, baseline cognitive impairment, major critical surgery) are warranted to explore the protective effect of ciprofol in these high-risk groups. At the mechanistic level, research integrating biomarker assays and neuroimaging techniques is essential to elucidate ciprofol's impact on neuroinflammation, blood-brain barrier function, and neural network activity. Furthermore, real-world studies will provide crucial evidence regarding the long-term benefits and cost-effectiveness of ciprofol.

## 5 Conclusion

This meta-analysis indicates that ciprofol may significantly reduce the risk of postoperative delirium in surgical patients compared to propofol. This protective effect appears particularly pronounced in patients undergoing major trunk surgery and those receiving total intravenous anesthesia. Despite the existing heterogeneity and potential biases in the current evidence, our findings provide valuable insights for clinical decision-making in perioperative neurocognitive protection. As a novel intravenous anesthetic, ciprofol holds considerable promise within the frameworks of enhanced recovery after surgery and precision anesthesia. Future large scale, high quality studies are warranted to further elucidate its neuroprotective mechanisms and definitive clinical value.

## Data Availability

The original contributions presented in the study are included in the article/supplementary material, further inquiries can be directed to the corresponding author.
